# Early-Phase Clinical Trials of Bio-Artificial Organ Technology: A Systematic Review of Ethical Issues

**DOI:** 10.3389/ti.2022.10751

**Published:** 2022-10-31

**Authors:** Dide de Jongh, Emma K. Massey, Antonia J. Cronin, Maartje H. N. Schermer, Eline M. Bunnik, the VANGUARD Consortium

**Affiliations:** ^1^ Department of Nephrology and Transplantation, Erasmus MC, University Medical Centre Rotterdam, Rotterdam, Netherlands; ^2^ Department of Medical Ethics, Philosophy and History of Medicine, Erasmus MC, University Medical Centre Rotterdam, Rotterdam, Netherlands; ^3^ Guy’s and St. Thomas’ NHS Foundation Trust, London, United Kingdom; ^4^ King’s College, London, United Kingdom

**Keywords:** ethics, regenerative medicine, bioengineering, research ethics, first-in-human clinical trials, bio-artificial organs, clinical trials, early-phase clinical trials

## Abstract

Regenerative medicine has emerged as a novel alternative solution to organ failure which circumvents the issue of organ shortage. In preclinical research settings bio-artificial organs are being developed. It is anticipated that eventually it will be possible to launch first-in-human transplantation trials to test safety and efficacy in human recipients. In early-phase transplantation trials, however, research participants could be exposed to serious risks, such as toxicity, infections and tumorigenesis. So far, there is no ethical guidance for the safe and responsible design and conduct of early-phase clinical trials of bio-artificial organs. Therefore, research ethics review committees will need to look to related adjacent fields of research, including for example cell-based therapy, for guidance. In this systematic review, we examined the literature on early-phase clinical trials in these adjacent fields and undertook a thematic analysis of relevant ethical points to consider for early-phase clinical trials of transplantable bio-artificial organs. Six themes were identified: cell source, risk-benefit assessment, patient selection, trial design, informed consent, and oversight and accountability. Further empirical research is needed to provide insight in patient perspectives, as this may serve as valuable input in determining the conditions for ethically responsible and acceptable early clinical development of bio-artificial organs.

## Graphical Abstract

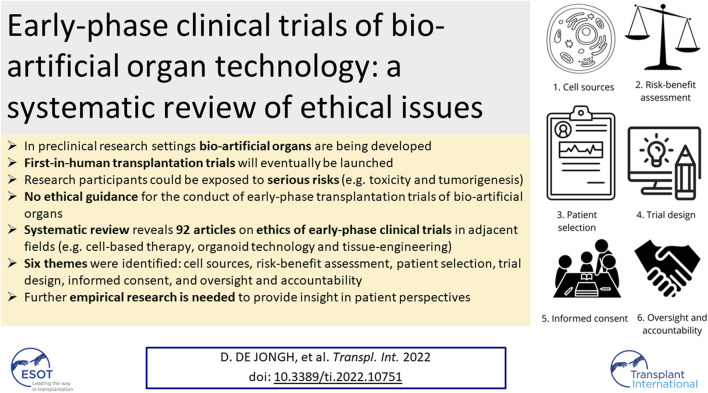

## Introduction

For patients with end-stage organ failure, having an organ transplant is often the best and only cure. Advances in surgical techniques and immunosuppressive medication means that organ transplantation is now widely and successfully used. However, there are still important challenges to overcome, notably the shortage of donor organs and the short and long-term side effects of taking lifelong immunosuppressive medication.

In the last decade, the multi-disciplinary field of regenerative medicine has emerged. Regenerative medicine uses technologies such as tissue engineering and 3D bioprinting to (re)generate, repair or replace damaged tissues and organs. Regenerative medicine and tissue engineering are terms often used interchangeably in the scientific literature. In this article however we use the term regenerative medicine to refer to the aim of the intervention (to regenerate), and tissue engineering to refer to the method for creating regenerative products. Regenerative medicine could, by way of illustration, combine patient-derived cells (e.g., in the form of organoids made from induced pluripotent stem cells) with cutting-edge technologies such as tissue engineering, to develop transplantable personalized bio-artificial organs. For example, the European Commission-funded VANGUARD project aims to engineer a vascularized and immune-protected bio-artificial pancreas for transplantation into patients with Type I Diabetes. The ambition of the VANGUARD project^1^ is for the transplanted bio-artificial pancreas to produce insulin and treat the underlying diabetic disease without requiring the patient to take lifelong immunosuppressive medication. Similarly, in other disease areas, first steps are being taken towards the generation of transplantable bio-artificial organs, including livers (1), bladders (2), kidneys (3), hearts (4), small intestines (5) and lungs (6, 7). These bio-artificial organs are currently still at the preclinical stage and are being tested in laboratory settings or animal studies.

It is likely that researchers will reach a point at which sufficient preclinical evidence has been collected to suggest that bio-artificial organs might be beneficial and safe for humans. At that point, early-phase clinical trials will be initiated to test the safety and efficacy of these products in humans. In early-phase clinical trials, human research participants could be exposed to serious risks, such as toxicity, infections and tumorigenesis. This is especially so in regenerative medicine trials requiring invasive and non-reversible procedures, resulting in permanent alterations of participants’ bodies (8).

It is not clear to what extent existing ethics oversight and guidance for the conduct of clinical trials is applicable to or sufficient for the clinical translation of bio-artificial organs. First, drug authorities, including the US Food and Drug Administration (FDA) and the European Medicines Agency (EMA), were originally set up to decide on marketing authorisation of *pharmaceutical agents*, not complex cell-based products. In Europe, bio-artificial organs are likely to be classified as Advanced Therapy Medicinal Products (ATMPs) (9), just like cell-based therapies. However, this classification may not completely cover the bio-artificial organ as, unlike most pharmaceutical agents, it is not a substance that can be injected or infused, but a complex product—more like a (cell-based) device—to be used in transplantation, which involves a (innovative) surgical intervention. Second, while there are internationally recognised guidelines for the ethical conduct of research involving human subjects, issued for instance by the Council for international Organization of Medical Science (CIOMS) (10) and the World Medical Association (WMA) (11), these guidelines should be expanded in order to make them applicable to the clinical translation of bio-artificial organs. The ethics guidelines of the International Society for Stem Cell Research (ISSCR) have been developed specifically for human stem cell research and clinical translation of cell-based interventions (12), but do not discuss applications of regenerative medicine in *organ transplantation*. Without the relevant guidance, it would be difficult for research ethics review committees (RECs) to evaluate the ethical acceptability of early-phase clinical trials of bio-artificial organs. Therefore, guidance on the safe and responsible design and conduct of early-phase clinical trials of transplantable bio-artificial organs should be developed.

In this systematic review we examined the published literature on early-phase clinical trials in the adjacent fields of regenerative medicine, including tissue-engineering, 3D bioprinting, cell-based therapy, organoid technology and synthetic biology. We undertook a thematic analysis of relevant ethical points to consider for early-phase clinical trials of transplantable bio-artificial organs. The results of our systematic review and thematic analysis will be valuable for researchers, research ethics review boards, policy makers and clinicians with an interest in regenerative medicine and involved in the translation of bio-artificial organs for clinical transplantation. However, above we hope our analysis will contribute to the preparation of robust guidelines and recommendations in this highly complex and evolving field.

## Methods

**FIGURE 1:**
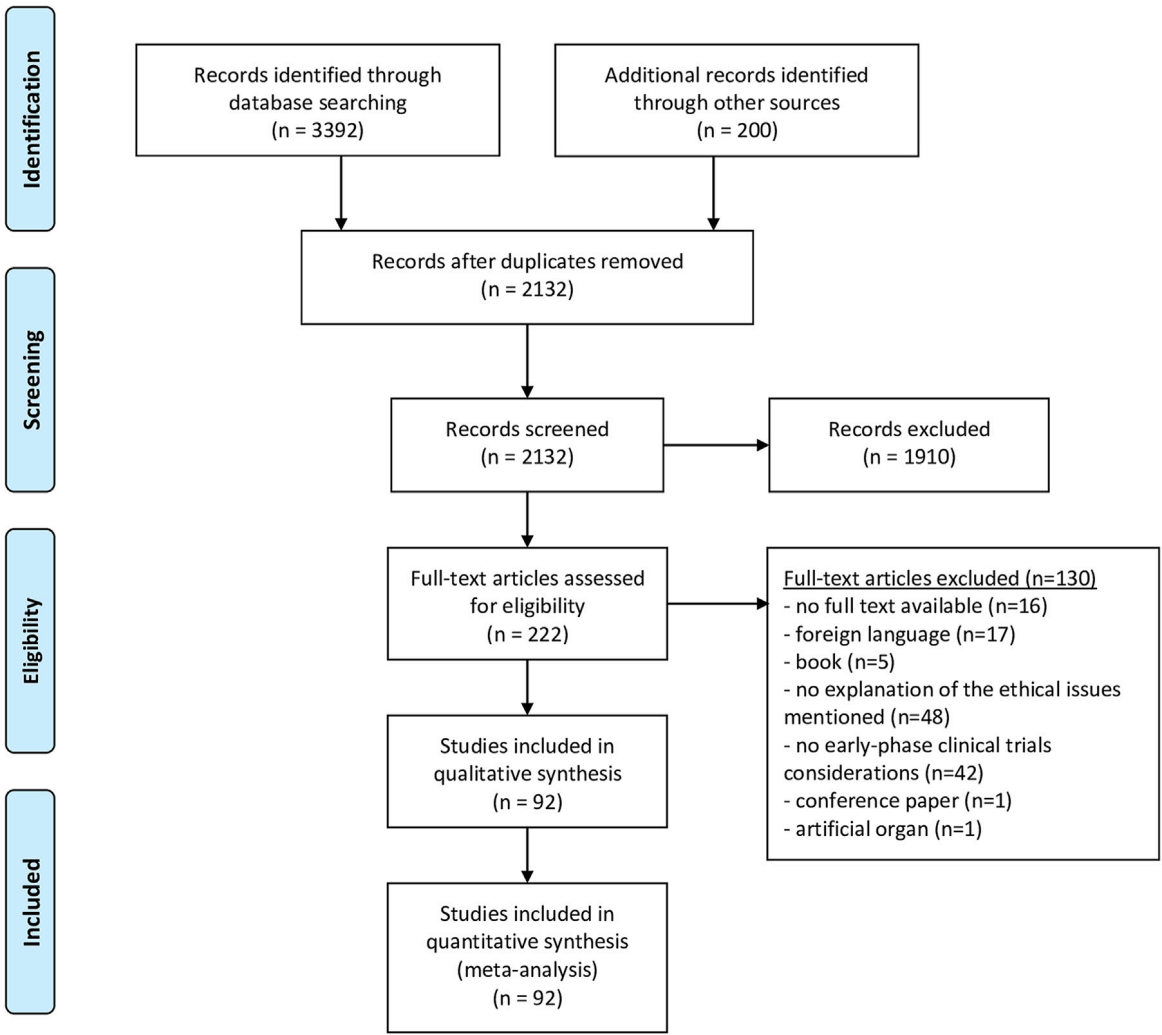
PRISMA flow diagram.

We performed a systematic review of the literature, following the PRISMA statement, as far as applicable (see Supplementary Materials). The review protocol has not been published or registered. The authors (DJ, EB and EM) developed the search strategy in consultation with a university librarian. We conducted the literature search in September 2021, using seven scientific databases: PubMed, EMBASE, Medline, Web of Science Core Collection, Cochrane Central Register of Controlled Trials and PsycINFO. An additional systematic search of the grey literature (i.e., relevant literature published outside of commercial or academic publishing) was conducted in Google Scholar. Search strings were constructed by keywords and their truncation, and relevant database-specific subjects headings [MeSH terms] (see Supplementary Materials). Due to language barriers, only articles in English or Dutch were considered for full-text analysis. We screened all titles and abstracts until September 2021 with no restriction for date of publication. Only outdated research guidelines that have subsequently been updated were not included. Based on title and abstract, articles that fulfilled the inclusion criteria were selected. Two researches independently carried out the selection (DJ and EB). Articles were discussed in case of differences between DJ and EB in the selection to come to a consensus. Full-texts were screened by DJ. The articles that did not meet the inclusion criteria during full-text screening, were excluded. Finally, the reference lists of the articles selected for full-text screening were checked for scientific articles or other documents that may be relevant and included if inclusion criteria were fulfilled (by DJ) (see Figure 1).

### Inclusion and Exclusion Criteria

The inclusion criteria of this systematic review were as follows: articles in the adjacent fields of regenerative medicine, tissue-engineering, 3D bioprinting, cell-based therapy, organoid technology, synthetic biology, and bio-artificial organs describing ethical points to consider (issues, questions or challenges) for early-phase clinical trials. Letters to the editor, editorials and opinion articles were included as non-research manuscripts. Articles that only discussed pre-clinical research were excluded from our sample. For reasons of feasibility, articles discussing transplantation of non-biological medical devices instead of biological materials (e.g., pacemakers, blood glucose monitors, insulin pumps, or cardioverter defibrators) and articles discussing engineering of specific tissues for purposes other than organ transplantation (e.g., engineering of brains and reproductive organs for research purposes) were excluded. Finally, conference abstracts and articles were excluded (Table 1).

**TABLE 1:** Inclusion and exclusion criteria.

Inclusion criteria	Exclusion criteria
Articles in the fields of regenerative medicine, tissue-engineering, 3D printing, cell-based therapy, organoid technology, synthetic biology and bio-artificial organs describing ethical points to consider (issues, questions, or challenges) for early-phase clinical trials	Letters to the editor
	Editorials
	Opinion articles
	Non-biological medical devices
	Engineering a specific tissue only for research purpose
	Describing ethical issues associated with pre-clinical research only

### Analyses and Syntheses

The method of qualitative content analysis was employed (13). Qualitative content analysis is an inductive (bottom-up) approach to categorize ethical considerations and to develop themes within a coding frame. One researcher (DJ) conducted the analyses. Firstly, codes were assigned to all the considerations mentioned in each publication. Secondly, themes (e.g., patient selection) were created out of these codes by DJ. Thirdly, DJ, EM and EB discussed whether the created words describing the themes were representative of the codes until agreement was reached. Finally, a coding framework was built out of the identified themes*.* The coding framework was used to systematically keep track of ethical considerations mentioned per article.

### Qualitative Content Analysis

We did not conduct a quality appraisal procedure, as there are no suitable criteria for appraisal of the quality of the literature included. This is a well-documented limitation of systematic reviews of (bio) ethical literature (14, 15).

## Results

The selection procedure is presented in a PRISMA Flow diagram (Figure 1). The search produced 2132 hits, of which 222 were deemed eligible on the basis of title and abstract, and 92 articles were included after reference checking and full-text screening. The publication dates ranged from January 2003 to March 2021 (Table 2).

**TABLE 2:** Included articles.

Author	Title	Year	Journal	Research field
Aalto-Setälä et al.	Obtaining consent for future research with induced pluripotent cells: opportunities and challenges	2009	PLoS Biology	Cell-Based Therapy
Afshar et al.	Ethics of research on stem cells and regenerative medicine: ethical guidelines in the Islamic Republic of Iran	2020	Stem Cell Research & Therapy	Regenerative Medicine
No Author	European Medicines Agency, CAR Secretariat and US Food and Drug Administration	2011	Regenerative Medicine	Cell-Based Therapy
Apatoff et al.	Autologous stem cell therapy for inherited and acquired retinal disease	2017	Regenerative Medicine	Cell-Based Therapy
**Attico et al.**	Approaches for effective clinical application of stem cell transplantation	2018	Current Transplantation Reports	Cell-Based Therapy
**Baker et al.**	Ethical considerations in Tissue Engineering Research: case Studies in Translation	2016	Methods	Tissue Engineering
Bhangra et al.	Using Stem Cells to Grow Artificial Tissue for Peripheral Nerve Repair	2016	Stem Cells International	Cell-Based Therapy
Bliss et al.	Optimizing the Success of Cell Transplantation Therapy for stroke	2010	Neurobiology of Disease	Cell-Based Therapy
Bobba et al.	The current state of stem cell therapy for ocular disease	2018	Experimental Eye Research	Cell-Based Therapy
**Bredenoord et al.**	Human tissues in a dish: The research and ethical implication of organoid technology	2017	Science	Organoid Transplantation
Brignier et al.	Embryonic and adult stem cell therapy	2010	Journal of Allergy and Clinical Immunology	Cell-Based Therapy
Chan.	Current and emerging global themes in the bioethics of regenerative medicine: the tangled web of stem cell translation	2017	Regenerative Medicine	Cell-Based Therapy
Chan.	Research Translation and Emerging Health Technologies: Synthetic Biology and Beyond	2018	Health Care Anal	Synthetic Biology
Chung	Stem-cell-based Therapy in the field of urology: a review of stem cell basic science, clinical application and future directions in the treatment of various sexual and urinary conditions	2015	Expert Opinion in Biological Therapy	Cell-Based Therapy
Coombe et al.	Current approaches in regenerative medicine for the treatment of diabetes: introducing CRISPR/CAS9 technology and the case for non-embryonic stem cell therapy	2018	American Journal Stem Cells	Cell-Based Therapy
Court et al.	Bioartificial liver support devices: historical perspectives	2003	ANZ Journal of Surgery	Bioengineered Organs
**Daley et al.**	Setting Global Standards for Stem Cell Research and Clinical Translation: The 2016 ISSCR Guidelines	2016	Stem Cell Reports	Cell-Based Therapy
Davis et al.	The role of Stem Cells for Reconstructing the Lower Urinary Tracts	2018	Current Stem cell Research & Therapy	Cell-Based Therapy
Davidson.	Brave Pioneers or Clinical Cowboys?	2010	Cell Stem Cell	Cell-Based Therapy
**De Vries et al.**	Ethical Aspects of Tissue Engineering: A Review	2008	Tissue engineering	Tissue Engineering
**De Windt et al.**	Ethics in musculoskeletal regenerative medicine; guidance in choosing the appropriate comparator in clinical trials	2019	Osteoarthritis and Cartilage	Regenerative Medicine
Fears et al.	Inclusivity and diversity: Integrating international perspectives on stem cell challenges and potential	2021	Stem Cell Reports	Cell-Based Therapy
Fung et al.	Responsible Translation of Stem Cell Research: An Assessment of Clinical Trial Registration and Publications	2017	Stem Cell Reports	Cell-Based Therapy
Garg et al.	Stem Cell Therapies in Retinal Disorders	2017	Cells	Cell-Based Therapy
**Genske et al.**	Rethinking risk assessment for emerging technology first-in-human trials	2016	Medicine, Health Care and Philosophy	Synthetic Biology
Giancola et al.	Cell therapy: cGMP Facilities and manufacturing	2012	Muscles, Ligaments and Tendons Journal	Cell-Based Therapy
**Gilbert et al.**	Print Me an Organ? Ethical and Regulatory Issues Emerging from 3D Bioprinting in Medicine	2018	Science and Engineering Ethics	3D Bioprinting
Goula et al.	Advanced Therapy Medicinal Products Challenges and Perspectives in Regenerative Medicine	2020	Journal of Clinical Medicine Research	Regenerative Medicine
Haake et al.	Concise Review: Towards the Clinical Translation of Induced Pluripotent Stem Cell-Derived Blood Cells- *Ready for Take-Off*	2019	Stem Cells Translational Medicine	Cell-Based Therapy
**Habets et al.**	The inherent ethical challenge of first-in-human pluripotent stem cell trials	2014	Regenerative Medicine	Cell-Based Therapy
**Hara et al.**	New Governmental Regulatory System for Stem Cell-Based Therapies in Japan	2014	Therapeutic Innovation & Regulatory Science	Cell-Based Therapy
Hayakawa et al.	A study on ensuring the quality and safety of pharmaceuticals and medical devices derived from the processing of allogeneic human somatic stem cells	2015	Regenerative Therapy	Cell-Based Therapy
Hildebrandt	Horses for courses: an approach to the qualification of clinical trial sites and investigators in ATMPs	2020	Drug Discovery Today	Cell-Based Therapy
Hug	Understanding voluntariness of consent in first-in-human cell therapy trials	2020	Regenerative Medicine	Cell-Based Therapy
**Hyun**	Allowing innovative Stem Cell-Based Therapies Outside of Clinical Trials: Ethical and Policy Challenges	2010	Journal of Law, Medicine and Ethics	Cell-Based Therapy
**Hyun et al.**	New ISSCR Guidelines Underscore Major Principles for Responsible Translational Stem Cell Research	2008	Cell Stem Cell	Cell-Based Therapy
Kim et al.	Report of the International Stem Cell Banking Initiative Workshop Activity: Current Hurdles and Progress in Seed-Stock Banking of Human Pluripotent Stem cells	2017	Stem Cells Translational Medicine	Cell-Based Therapy
**King et al.**	Ethical issues in stem cell research and therapy	2014	Stem Cell Research & Therapy	Cell-Based Therapy
Kleiderman et al.	Overcoming barriers to facilitate the regulation of multi-centre regenerative medicine clinical trials	2018	Stem Cell Research & Therapy	Regenerative Medicine
Knoepfler	From Bench to FDA to Bedside: US Regulatory Trends for New Stem Cell Therapies	2015	Advanced Drug Delivery Reviews	Cell-Based Therapy
**Kusunose et al.**	Informed consent in clinical trials using stem cells: Suggestions and points of attention from informed consent training workshops in Japan	2015	South African Journal of Bioethics and Law	Cell-Based Therapy
Lederer et al.	Neural stem cells: mechanisms of fate specification and nuclear reprogramming in regenerative medicine	2008	Biotechnology Journal	Cell-Based Therapy
Lee et al.	Conditional approvals for autologous stem cell-based interventions	2018	Perspectives in Biology and Medicine	Cell-Based Therapy
Levin et al.	Special Commentary: early Clinical Development of Cell Replacement Therapy: Considerations for the National Eye Institute Audacious Goals Initiative	2017	Ophthalmology	Cell-Based Therapy
Lim et al.	Whole Organ and Tissue Reconstruction in Thoracic Regenerative Surgery	2013	Mayo clinic Proceedings	Tissue Engineering
Liras	Future research and therapeutic applications of human stem cells: general, regulatory, and bioethical aspects	2010	Journal of translational Medicine	Cell-Based Therapy
Liu et al.	Advances in Pluripotent Stem Cells: History, Mechanisms, Technologies, And Applications§	2020	Stem Cell Reviews and Reports	Cell-Based Therapy
Lomax et al.	Return of results in translational iPS cell research: considerations for donor informed consent	2013	Stem Cell Research & Therapy	Cell-Based Therapy
Lomax et al.	Regulated, reliable and reputable: Protect patients with uniform standards for stem cell treatments	2020	Stem Cells Translational Medicine	Cell-Based Therapy
Lowenthal et al.	Specimen Collection for Induced Pluripotent Stem Cell Research: Harmonizing the Approach to Informed Consent	2012	Stem Cells Translational Medicine	Cell-Based Therapy
**Lowenthal et al.**	Ethics and Policy Issues for Stem Cell Research and Pulmonary Medicine	2014	Chest	Cell-Based Therapy
Lu et al.	Tissue Engineered Constructs: Perspectives on Clinical Translation	2015	Annals of Biomedical Engineering	Tissue Engineering
Madariaga et al.	Bioengineering Kidneys for Transplantation	2014	Seminars in Nephrology	Bioengineered Organs
Maekawa et al.	Development of Novel Advanced Cell and Gene Therapy and GMP-Controlled Cell Processing	2005	Japan Medical Association journal	Cell-Based Therapy
**Main et al.**	Managing the potential and pitfalls during clinical translation of emerging stem cell therapies	2014	Clinical and Translational Medicine	Cell-Based Therapy
Masuda et al.	New Challenges for Intervertebral Disc Treatment Using Regenerative Medicine	2010	Tissue engineering	Regenerative Medicine
**Moradi et al.**	Research and therapy with induced pluripotent stem cells (iPSCs): Social, legal and ethical considerations	2019	Stem Cell Research & Therapy	Cell-Based Therapy
Nagamura	The Importance of Recruiting a Diverse Population for Stem Cell Clinical Trials	2016	Current Stem Cell Reports	Cell-Based Therapy
Naghieh et al.	Biofabrication Strategies for Musculoskeletal Disorders: Evolution towards Clinical Application	2021	Bioengineering	3D Bioprinting
**Nagpal et al.**	PERSPECTIVES: Stroke survivors’ views on the design of an early‐phase cell therapy trial for patients with chronic ischaemic stroke	2019	Health Expectations	Cell-Based Therapy
Neri	Genetic Stability of Mesenchymal Stromal Cells for Regenerative Medicine Applications: A Fundamental Biosafety Aspect	2019	International Journal of Molecular Sciences	Cell-Based Therapy
**Niemansburg et al.**	Participant selection for preventive Regenerative Medicine trials: ethical challenges of selecting individuals at risk	2015	Journal of Medical ethics	Regenerative Medicine
**Niemansburg et al.**	Regenerative medicine interventions for orthopedic disorders: ethical issues in the translation into patient	2013	Regenerative Medicine	Regenerative Medicine
**Niemansburg et al.**	Ethical implications of regenerative medicine in orthopedics: an empirical study with surgeons and scientists in the field	2014	The spine Journal	Regenerative Medicine
**O’Donnell et al.**	Beyond the Present Constraints That Prevent a Wide Spread of Tissue Engineering and Regenerative Medicine Approaches	2019	Frontiers Bioengineering and Biotechnology	Regenerative Medicine
**Oerlemans et al.**	Regenerative Urology Clinical Trials: An Ethical Assessment of Road Blocks and Solution	2013	Tissue engineering	Tissue Engineering
**Oerlemans et al.**	Towards a Richer Debate on Tissue Engineering: A Consideration on the Basis of NEST-Ethics	2012	Science Engineering Ethics	Tissue Engineering
O’Keefe	American Society for Bone and Mineral Research- Orthopaedic Research Society Joint Task Force Report on Cell-Based Therapies	2020	Journal of Bone and Mineral Research	Cell-Based Therapy
**Otto et al.**	Ethical considerations in the translation of biofabrication technologies into clinic and society	2016	Biofabrication	3D Bioprinting
Parent et al.	The ethics of testing and research of manufactured organs on brain-dead/recently deceased subjects	2019	Journal of Medical Ethics	Bioengineered Organs
Patuzzo et al.	3D bioprinting Technology: Scientific Aspects and Ethical Issues	2018	Science and Engineering Ethics	3D Bioprinting
**Schneemann et al.**	Ethical challenges for pediatric liver organoid transplantation	2020	Science Translational Medicine	Organoid Transplantation
Scopetti et al.	Mesenchymal stem cells in neurodegenerative diseases: Opinion review on ethical dilemmas	2020	World Journal of Stem Cells	Cell-Based Therapy
Sekar et al.	Current standards and ethical landscape of engineered issues—3D bioprinting perspective	2021	Journal of Tissue Engineering	3D Bioprinting
Seok et al.	A Personalized 3D-Printed Model for Obtaining Informed	2021	Journal of Personalized Medicine	3D Bioprinting
	Consent Process for Thyroid Surgery: A Randomized Clinical Study Using a Deep Learning Approach with Mesh-Type 3D Modeling			
**Shineha et al.**	A Comparative Analysis of Attitudes on Communication Toward Stem Cell Research and Regenerative Medicine Between the Public and the Scientific Community	2018	Stem Cells Translational Medicine	Regenerative Medicine
Sievert et al.	Tissue Engineering for the Lower Urinary Tract: A Review of a State of the Art Approach	2007	European Urology	Tissue Engineering
Smith et al.	Challenging misinformation and engaging patients: characterizing a regenerative medicine consult service	2020	Regenerative Medicine	Regenerative Medicine
Sniecinski et al.	Emerging stem cell based strategies for treatment of childhood disease	2018	Transfusion and Apheresis Science	Cell-Based Therapy
Stegemann et al.	Cell therapy for bone repair: narrowing the gap between vision and practice	2014	European Cells and Materials	Cell-based therapy
Sugarman and Bredenoord	Real-time ethics engagement in biomedical research	2020	EMBO reports	Organoid transplantation
Sutherland and Mayer	Ethical and Regulatory Issues Concerning Engineered Tissues for Congenital Heart Repair	2003	Thoracic and Cardiovascular Surgery	Tissue Engineering
**Takashima et al.**	Lessons for reviewing clinical trials using induced pluripotent stem cells: examining the case of a first-in-human trial for age-related macular degeneration	2018	Regenerative Medicine	Cell-Based Therapy
**Taylor et al.**	Ethics of bioengineering organs and tissues	2014	Expert Opinion on Biological Therapy	Tissue Engineering
**Trommelmans et al.**	Ethical reflections on clinical trials with human tissue engineered products	2008	Journal of Medical Ethics	Tissue Engineering
**Trommelmans et al.**	Informing participants in clinical trials with *ex vivo* human tissue-engineered products: what to tell and how to tell it?	2008	Journal Tissue Engineering Regenerative Medicine	Tissue Engineering
Trommelmans et al.	An Exploratory Survey on the Views of European Tissue Engineers Concerning the Ethical Issues of Tissue Engineering Research	2009	Tissue Engineering	Tissue Engineering
**Trommelmans et al.**	Is tissue engineering a new paradigm in medicine? Consequences for the ethical evaluation of tissue engineering research	2009	Medical Health Care and Philosophy	Tissue Engineering
Tsang	Legal and ethical status of stem cells as medicinal products	2005	Advanced Drug Delivery	Cell-Based Therapy
**Vijayavenkataraman et al.**	3D bioprinting - An Ethical, Legal and Social Aspects (ELSA) framework	2016	Bioprinting	3D Bioprinting
Zamborsky et al.	Regenerative Medicine in Orthopaedics and Trauma: Challenges, Regulation and Ethical Issues	2018	Orthopaedics and Trauma	Cell-Based Therapy
Zocchi et al.	Regulatory, ethical, and technical considerations on regenerative technologies and adipose-derived mesenchymal stem cells	2019	European Journal of Plastic Surgery	Regenerative Medicine

^a^ Author name stated in **bold**: ethical considerations for early-phase regenerative trials are elaborately discussed in the paper.

### Themes

**FIGURE 2:**
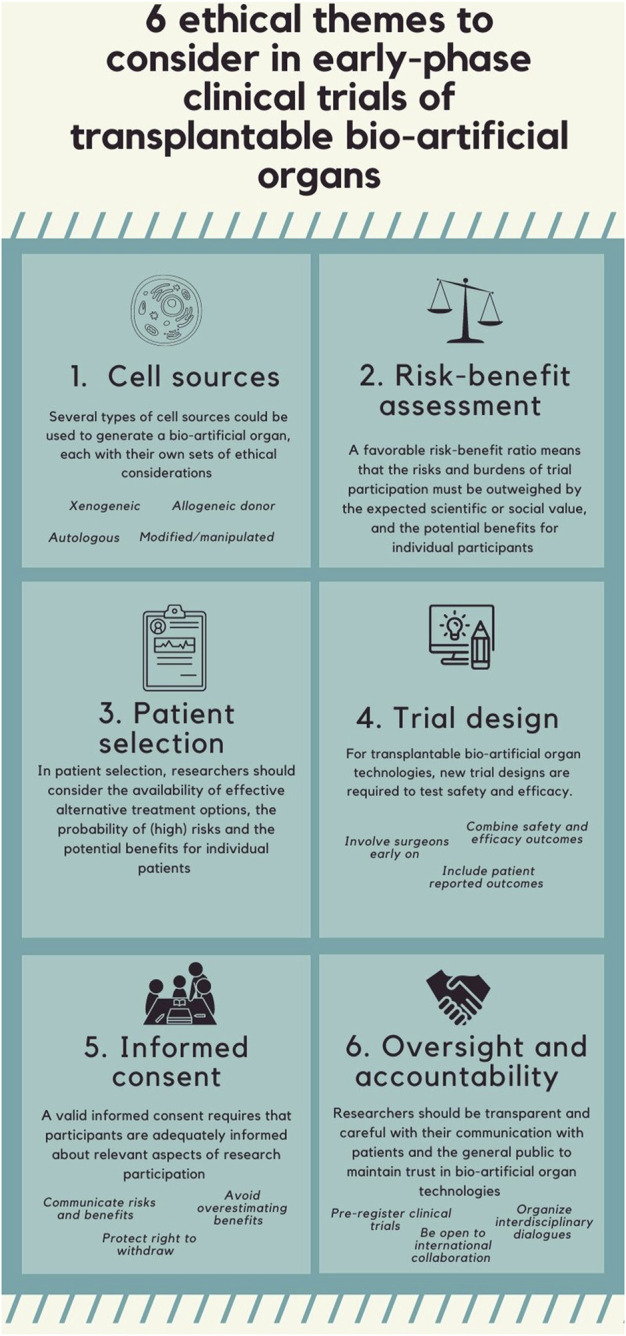
Summary of the content of the article.

Six themes were identified: cell source, risk-benefit assessment, patient selection, trial design, informed consent, and oversight and accountability. The content of the article referring to the six identified ethical themes is summarized in Figure 2.

### Research Fields

These six themes were found in seven different research fields (Table 2). The largest body of literature focusses on ethical considerations around early-phase trials in the field of cell-based therapy; 55 articles are published in this field, and the authoritative ISSCR guidelines are widely used (12, 16–26). There is less literature on ethical aspects of early-phase clinical trials in the field of 3D bioprinting, and organoid transplantation; seven articles were published on 3D bioprinting, three articles on bio-artificial organs, and two on organoid transplantation. Six empirical studies using questionnaires and interviews to investigate patients’ and professionals’ views on ethical considerations in early-phase clinical trials, were included. Seven papers were published in surgical journals.

### Theme 1: Cell Sources

53 out of 92 articles mention ethical considerations related to the sources of cells used to generate complex tissue-engineered products such as bio-artificial or 3D bio-printed organs for transplantation into humans (9, 12, 16–24, 26–68). There are four types of cell sources: 1) xenogeneic cells, 2) autologous, 3) allogeneic donor, and 4) highly manipulated or/and genetically modified cells in humans, each with their own sets of ethical considerations (Table 3).

**TABLE 3:** Points to consider in relation to cell sources.

Cell source	Risks and benefits	Points to consider
Xenogeneic cells or tissue	Medical risks: Risk of zoonoses Individuals could object to use cells derived from animals on religious or socio-cultural grounds	- The use of animal cells should be minimized
		- Components of animal origin should be replaced with human or chemically defined components whenever possible
		- The use of viral transcription factor genes, retroviruses or pathogenic agents should be minimized
		- Quality control systems, standard operating procedures (SOPs) and Good Manufacturing Practice (GMP) should be used
Autologous cells	Medical benefits: No immunological rejection	- It may not be possible to harvest sufficient numbers of patients’ cells
		- The production cost could be high
		- The timeframe for cell harvest could be insufficient for timely treatment
		- Extra surgical interventions for participants could be necessary
		- Quality control systems, SOPs and GMP should be used
Allogeneic donor cells	Medical risks: Immunological rejection and disease transmission	- Adequate donor consent should be obtained in a process that includes discussion of: aim of the research, return of research results, incidental findings, possibilities for withdrawal of consent, potential future research
		- Additional safeguards should be adopted to protect personal data
	Relational issues: Ownership and privacy issues Some donors may not want their cells to become an integral, growing part of another person.	- A policy should be developed on whether and how incidental findings of donor cell (genetic) screening should be returned to the cell donors and/or their relatives
		- Records on medical and family history of the donor of the cells should be obtained periodically
		- Quality control systems, SOPs and GMP should be used
Highly manipulated and/or genetic modified cells	Medical risks: Unexpected behavior of cells or tissue (e.g., tumor formation, epigenetic or genetic instability)	- Strong pre-clinical data (of the safety and functions of the cells and or tissues) should be provided
		- The use of manipulated cells should be minimized
		- Participants should be monitored for a long time
		- Researchers should adhere to cell processing and manufacturing protocols
		- Quality control systems, SOPs and GMP should be used

Firstly, xenogeneic cells are associated with a risk of zoonosis (17, 20, 38, 47–49). For instance, issues related to the transmission of the infectious porcine retrovirus (PERV) from pig to human (69). Potential future patients could also reject the use of these cells to generate bio-artificial organs on religious grounds or for socio-cultural reasons (e.g., to protect animal rights/welfare) (33, 38, 48, 50, 52), even if their religious leaders take a more moderate stance (33). According to the literature, using these cells for transplantation into humans should be minimized as much as possible (12, 17, 38).

Secondly, the use of autologous cells (cells taken from the patient, who is both the donor and recipient) will make immunosuppressive therapy unnecessary (9, 16, 27–29, 33, 38–45, 68), and is perceived to carry fewer risks than the use of other cell types (33). However, challenges include the high production costs (29, 57, 70), extra surgical interventions for participants (50), the time required for their production (29, 40, 50, 57, 70), and the difficulty of standardizing manufacturing procedures (40 57, 70).

Thirdly, besides the medical risks of transplanting allogeneic donor cell (cells taken from another human being), for example developing immunological problems, use of these cells also raises relational issues (20, 27, 30, 38, 41, 43, 63, 71, 72). Relational issues include questions such as: Who is the owner of the human cells once it is separated from the body (30,38,41,43)?; Can cells from the human body be subjected to laws regarding property rights (38,43)?, and; To what extent can the donor’s privacy and confidentiality be ensured by adopting additional measures (e.g., pseudonymisation) (20, 27, 30, 38, 41, 43, 63, 71, 72). Removing the donor’s personal information is often not desirable, because subsequent research may necessitate ongoing access to the information about the cell donor’s health status requiring personal data of the donor (e.g., their name and/or address) (20,52). Further, some donors may not want their cells to become an integral, growing part of another person (12, 20, 32, 52, 73). In addition, in the course of donor cell (genetic) screening, researchers should develop a policy on whether and how incidental findings (e.g., genetic risk) will be returned to the donors and/or their relatives (12, 20, 52, 63). Donors might consider their privacy violated if scientists know their future susceptibility to genetic disorders (52). Researchers should obtain an adequate informed consent from donors to respect their autonomy (12, 20, 22, 27, 28, 34, 38, 43, 45, 52, 57, 63, 67, 72–76), and give them some degree of insight and perhaps control over the use of donated materials by informing them about the types of incidental findings they wish to receive, future commercial applications, individualized research and therapeutic uses (12, 20, 27, 38, 43, 52, 72, 76), for instance by maintaining an ongoing dialogue with the donors (76). Moreover, to safeguard the health of the recipient over the years, it may be necessary to periodically obtain records on the medical and family history of the cell donor to monitor potential health risks, such as long-term immunological or tumorigenic reactions (12, 19, 20, 22, 27, 28, 32, 34, 35, 39, 41, 49, 51–53).

Lastly, the use of highly manipulated cells (i.e., cells of which the biological nature or structural function has been altered during the manufacturing process) and/or genetically modified cells raises safety concerns, and requires more quality controls to avoid undesired events (9, 12, 18, 20–23, 27, 28, 33, 35, 40, 50, 61, 63). For instance, these cells could have an increased risk of being tumorigenic, genetically unstable or toxic (12, 18, 35). Therefore, some authors recommend avoiding the use of manipulated cells whenever possible (e.g., tumor formation, epigenetic or genetic instability) (9, 12, 18, 20, 22). However, cell manipulation and/or genetic modification might be useful and even necessary for the generation of a bio-artificial organ (e.g., to repair disease-causing mutations) (20). Cells used in tissue-engineered products are often differentiated *in vitro* prior to being combined with a scaffolding material, for example collagen, to form artificial tissue, therefore tissue-engineered products are mostly classified as more than minimally manipulated (18).

### Theme 2: Risk-Benefit Assessment

**FIGURE 3:**
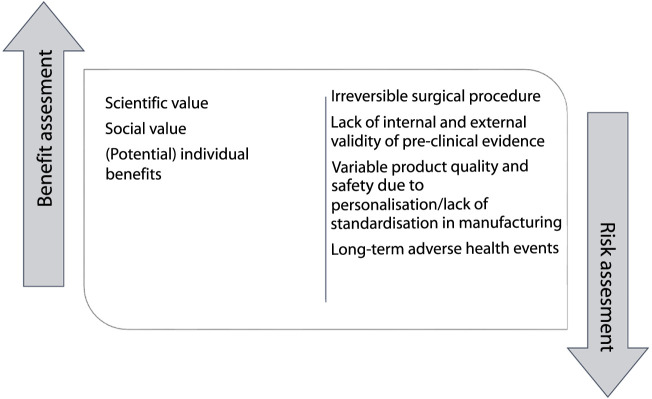
Risk-benefit assessment.

One of the conditions for ethically responsible clinical research is a favorable risk-benefit ratio (Table 4). This means that the risks and burdens of trial participation must be outweighed by the expected scientific or social value and the (potential) benefits for individual participants (12, 16, 21, 23, 24, 28, 29, 32, 34, 37, 45, 50, 53, 57, 64, 66–69, 77, 78) (Figure 3). The requirement of a favorable risk-benefit is difficult to meet in early-phase research, because the potential direct benefits to individual research participants in these trials are limited and uncertain (69). In the absence of direct medical benefit, justification of exposing individual research participants to potential harms in early-phase clinical trials is sought in expected scientific and/or social value (24, 30, 50, 66, 79). These include the benefits gained for science and society: generalizable knowledge and health gains for future patients (50). Knowledge of the working mechanism and the interaction of a regenerative medicine technology with the body, gathered in early-phase clinical trials, is necessary to move these technologies to the next clinical phase of clinical development (24, 30, 50, 66, 69). The anticipated social value of bio-artificial organs is potentially high, as they are intended as cures for patients with end-stage organ failure and might be more cost-effective than existing organ replacement therapies (66). At this stage, however, the social value is highly uncertain.

**TABLE 4:** Points to consider in relation to risk-benefit assessment.

Points to consider in relation to risk-benefit assessment
- Researchers should provide robust pre-clinical data (i.e. safety and efficacy of the product should be rigorously demonstrated in laboratory tests and animal models)
- Personalization of the bio-artificial organ makes the product variable; therefore, the quality control and safety requirements of mass manufacturing do not apply
- Researchers should monitor and follow up participants for a long time after the study
- Efforts should be made not only to minimize the risks, but also to maximize the scientific and social value of a trial, in order to improve the risk-benefit ratio
- Clinical teams who conduct clinical trials of bioartificial organs should have experience with regenerative medicine technologies and with post-trial follow-up care

Transplanting regenerative medicine into human recipients requires an irreversible (innovative) surgical procedure, which is associated with risks of harms and complications. Once the regenerative product is implanted in the body, it may not be possible to completely remove it (50). For instance, surgical removal of the product will be impractical or associated with greater risks [i.e., infections or complications of anesthesia (33)], and there will be some irreversible changes, such as scarring (50, 70). In addition, unlike non-biological medical devices, the regenerative product will most likely interact and integrate with the rest of the body, which may have uncertain, possibly unforeseeable long-term adverse health events for the recipient (16, 18, 21, 23, 24, 27, 28, 31–34, 37–40, 48, 50, 58, 62, 66–70, 72, 73, 77, 79–86).

When researchers are dealing with uncertain but potentially high risks, they are advised, before undertaking an early-phase clinical trial, to provide preclinical evidence of high internal validity (e.g., through replication) and external validity (e.g. through careful study design) (12, 16, 23, 27–29, 31, 34–37, 43, 46, 49–51, 53, 57, 59, 61, 62, 64–69, 77, 79–81, 84, 85, 87–90). Some argue that large animals should be used, because these animals can better imitate the human anatomy and/or pathology than small animals (12 81). Others recommend to involve unbiased third parties to repeat some of the research (69). Even if robust preclinical evidence is available using these strategies, some unexpected risk will inevitably remain, such as unforeseeable long-term adverse health events for the recipient. Researchers should be aware that preclinical evidence from animal models may not correctly predict the duration, function and interaction that occur in a human body (16, 24, 27, 31, 34, 37, 39, 50, 65, 68, 79–82). In addition, the personalization of regenerative medicine makes the product variable, therefore, the quality control and safety requirements of mass manufacturing for external validity do not apply (32, 34, 35, 48). A major benefit of personalization, however, is that it may take away or reduce the need for the use of life-long immunosuppressive therapy for recipients, and avoid well-known side effects such as infections and nephropathy (45, 69).

To detect health risks associated with potential long-term adverse events, such as genetic instability, undirected or uncontrolled cell growth, research participants must be carefully monitored (16, 19, 21, 23, 24, 28, 29, 32, 34, 42, 46, 50, 58, 64, 67–70, 81–83, 85), with long-term follow-up (12, 19, 21, 23, 27–29, 32, 34, 35, 37, 38, 40, 46, 50, 51, 53, 62, 66–70, 73, 79, 81, 85, 87, 91, 92). On the one hand, intensive monitoring may be perceived as reassuring or beneficial by research participants (50, 83, 93). On the other hand, possible life-long follow-up could also be burdensome for participants (50). Given the complexity of tissue-engineered products, clinical teams conducting these studies should have experience with other regenerative medicine therapies (e.g., cell-based therapy) and with post-trial follow-up care (81).

### Theme 3: Patient Selection

In the patient selection procedure, a new kind of trade-off has to be made: against enormous benefits stand potentially large risks (e.g., tumour formation). Selection of patients in early-phase clinical trials is a major ethical theme in the literature (12, 27, 31–34, 37, 42, 43, 45, 48, 50, 66, 67, 69, 70, 77, 81, 82, 94). Potential target groups can be divided into 5 categories: healthy individuals, individuals at risk, children, patient with early-stage disease and patients with end-stage disease (Table 5). First, it is considered unacceptable to ask 1) healthy individuals for clinical studies of regenerative medicine applications, especially of tissue-engineered products which are designed to function in the body of the recipient, given the high risks (34) and lack of benefit (32,34). Also, when regenerative applications are personalized (i.e., composed, in part, of patient-derived material), the only eligible recipient will likely be the patient themselves (48). Second, the scholarly literature contains arguments in favour of the selection of 2) individuals at risks, with 3) early-stage disease (31, 37, 48, 50, 69, 77, 81, 94), and 4) children (37, 38, 48, 78). These individuals are relatively healthy, if a regenerative medicine application is used into one of these groups, it may help 1) to achieve more health benefit, and 2) to prevent (long-term) severe complications (31, 37, 48, 50, 69, 77, 81, 94). On the other hand, it is uncertain whether these individuals, who may not have developed or will develop symptoms at all, will indeed come to suffer from end-stage organ failure at all and be in need for a transplant. At the same time, as the procedure is novel, risky and invasive, their current physical condition could worsen significantly (50). Lastly, based on the literature, the most eligible patients for early-phase clinical trials are patients who have reached the 5) end-stage of their disease (12, 27, 31, 33, 34, 42, 43, 45, 48, 66, 69, 70, 81, 82, 94). These patients no (or no longer) have effective or suitable treatment options at the time of enrolment and may be facing limited life expectancy (12, 27, 31, 33, 34, 42, 43, 45, 48, 66, 69, 70, 81, 82, 94). When serious complications occur, they may have less to lose than healthy individuals or patients with stable disease (, 12, 32–34, 48, 50, 66, 67, 77, 94). Also, for patients who have reached the end-stage of their disease, a bio-artificial organ could potentially be associated with greater medical benefits.

**TABLE 5:** Points to consider in relation to patient selection.

Suggested research participants for early-phase clinical trials	Reasons for and against selection
Healthy individuals	For
	- Healthy individuals are most resilient to physical harms (thus, harms are minimized)
	Against
	- No clinical value for the participant
	- Risks are too high
Individuals at risk	For
- No symptoms	- Less damage to the body from disease or disease-related complications, which could lead to better health outcomes compared to more advanced disease stages
- Risk factors for disease	- Disease can be prevented
	Against
	- Risks could be too high
	- Unnecessary treatment (participants may not develop the disease)
Early-stage patients	For
- Mild to moderate disease	- Less damage to the body from disease or disease-related complications, which could lead to better health outcomes compared to more advanced disease stages
- Medically controlled disease	Against
	- Risks are too high
	- Alternative treatment options may be available
	- Treatment could worsen the disease
Children	For
Diagnosed with the disease	- Less damage to the body
	- Serious complications can be prevented
	- Benefit can be enjoyed the longest
	Against
	- Risks may be too high
	- Alternative treatment options may be available
	- The disease may not proceed to advanced stages
	- Long-term follow-up may be burdensome for the participants
	- Children are unable to provide informed consent
Advanced-stage/end-stage patients	For
- Severe disease	- There is an unmet medical need, as effective treatment options are not or no longer available
- Unstable disease	- Potential for medical benefit from participation in the trial
- No or no longer a suitable treatment option available	- Less to lose when serious complications occur
	Against
	- The body is already damaged; this damage might be irreparable
	- Treatment could worsen the disease

### Theme 4: Trial Design

#### Intervention

Six articles in our sample argued that the traditional model for clinical translation—phases I to phases II, III and IV, in which toxicity and/or efficacy of new drugs are tested—may not be suitable for clinical trials of transplantable applications of regenerative medicine in humans (17, 24, 37, 38, 62, 81). Schneemann et al. proposed that early-phase transplantation trials should combine safety and efficacy outcomes in their trial design to maximise participants’ chances at obtaining medical benefit (37). Schneemann et al. suggested participants should be given a “dose” (in the context of bio-artificial organs: a certain quantity of engineered tissue) that is expected to be therapeutic, and efficacy should be added as an outcome measure (37). Combined safety and efficacy trials are associated with lower risks and costs than traditional studies, which could have positive effects on the likelihood of successful clinical development and help prevent promising interventions from failing (17, 81).

#### Outcomes

In the literature, relevant outcome measures for regenerative medicine clinical trials are discussed in 18 papers (12, 16, 19, 21, 24, 32, 34, 37, 43, 50, 61, 64, 69, 77, 80, 81, 87, 94). Both clinical outcome measures (e.g., survival rate or functional status) and patient-reported outcome measures (PROMs) (e.g., quality of life or experienced symptoms) are considered important (12, 21, 34, 43, 69, 77, 81, 87, 94). In later stages of clinical development and implementation, registries should be set up so that real-world outcome data can be collected to facilitate fair evaluation of the benefits of this technology. In addition, in later stages researchers should not only measure clinical outcome measures, but also PROMs, in order to ensure that new technologies not only affect biological parameters favourably, but also improve patients’ lives (37, 69, 94). By giving potential participants the opportunity to define outcome measures, they become active stakeholders in the trial design (37, 69, 78, 94). Further, asking patients to define outcomes could help increase the enrolment of participants in the trial (21, 37, 69, 94).

#### Skills and Materials

Authors also suggest to involve surgeons early on in the trial design, since they know what surgical skills and materials are needed to perform surgical trials safely (43, 37, 35, 12, 87). Clinical translation of bio-artificial organs in transplantation may require surgeons to learn new techniques and develop new instruments, therefore minimizing the number of surgeons involved is suggested (Table 6). Additionally, different surgeons may learn and refine surgical techniques in different ways, which may (temporarily) affect the outcomes of trials (34, 68, 95). Therefore, it is advised to account for a learning curve and for variability in experience between surgeons (32, 68, 66, 77, 96).

**TABLE 6:** points to consider in relation to trial design.

Trial design	Points to consider
Intervention	- Researchers should set up combined efficacy and safety trials
Outcomes	- Patients should be actively involved in research design as stakeholders
1. Patient-reported (e.g., quality of life, treatment satisfaction and experienced symptoms)	- PROMs should be developed for later-phase clinical trials and adopted in trial design
2. Professional defined (survival rate, functional status and biological parameters)	
Skills and materials	- Learning curves of surgeons should be corrected for
	- The effects of the risks associated with surgical procedures on the outcomes of trials should be corrected

### Theme 5: Informed Consent

The ethical requirements of clear informed consent is mentioned frequently in the literature (12, 16, 17, 20–25, 27, 29, 31–34, 37, 38, 43, 45, 50–52, 59, 60, 64–69, 75–77, 79, 81, 83, 85, 89, 90, 92, 93, 97, 98). Valid informed consent requires that participants must be adequately informed about relevant aspects of research participation, including the aim of the procedure, duration of the study, their right to withdraw, and the risks and benefits implications of the trial (Table 7). Less often mentioned as an essential component in informed consent is information on the specific composition of the regenerative medicine application, although some authors find it important (33, 81, 83). One survey showed that participants want to be especially informed about issues that could directly affect their health status, such as foreseeable risks, impact on quality of life and safety measures (83). Participants are worried about the risks associated with genetic manipulation of transplantable tissue and about commercialization of cells (33, 83).

**TABLE 7:** points to consider in relation to informed consent.

Procedural	Substantial
- Informed consent from participants with decisional capacity or their legally authorized	- Potential risks, benefits and uncertainties
representative should be obtained
- Relevant information about the trial, should also be presented visually	- Composition of the product
- Patients should be encouraged to ask questions	- The irreversible nature of the intervention
- Scientific jargon should be avoided by using only simple words or easily understood terminology	- How adverse events will be dealt with - The right and practical difficulty
- The teach-back method, exams or questionnaires could be used to ensure that participants	to withdraw
understand the relevant information	- How life-long follow up will be organized
- Participants should be encouraged to ask independent experts/patient advocates for advice or assistance in the decision-making process	- The possibility to consent for partial or complete autopsy in the event of death
- Participants need to be informed that the intervention is not likely to provide direct medical benefits	

Given the lack of evidence on the risks, however, it could be difficult for researchers to provide full disclosure. Rather, participants should be made aware of the uncertainties surrounding the risks and benefits of investigational regenerative medicine technologies (20, 21, 23, 24, 32–34, 65, 72, 81, 98). Participants should be given the opportunity to consult an independent expert (33, 98), and can be offered psychological support (81)**,** or consult a patient advocates (81)**,** to assist them in the decision-making process (33, 60, 81, 83, 84, 98). To minimize “the therapeutic misconception,” the (sometimes) mistaken belief among research participants that they will benefit from trial participation, measures should be taken to ensure that research participants are aware of the fact that research is conducted not with the goal of providing them medical treatment, but of obtaining generalizable information (12, 16, 17, 21, 24, 25, 29, 31, 33, 37, 50, 57, 60, 64, 67, 69, 81, 93, 97, 98). Researchers should avoid presenting the potential of the product in an overly optimistic light, overestimating the possible benefits, or giving unrealistic timelines for it to reach the clinic (30). Also, to strengthen comprehension, researchers are advised to present information about the trial not only in writing but also visually (33, 60, 68, 79), encourage patients to ask questions, and avoid scientific jargon by using only simple words or easily understood terminology during the informed consent process (20–22, 29, 31, 57, 69, 93, 98). Researchers may use the teach-back method (98) or even an “exam” or questionnaire (33) to ensure that participants understand the information and make an informed choice (33, 34, 81, 98, 99). Participants must also be aware that participating in a trial might diminish their chances of getting access to future treatment opportunities (21,48,50).

A widely endorsed norm in research ethics is that participants should always have the right to withdraw their consent without negative consequences for the health care they receive. However, for participants in early-phase clinical trials of regenerative medicine technologies, withdrawal may be complicated (34). While it may be possible to withdraw from follow-up, removal of bio-artificial organs (in their entirety) may not be possible. For this reason, the opportunities for withdrawal or lack thereof, and the implications of trial participation for the future health and safety of participants must be discussed beforehand, as part of the informed consent process (34). In particular, research participants should be aware of the need for a long-term follow-up and the possibility of (long-term) adverse events (32, 34, 81). Lastly, some authors suggest informing and asking participants to provide consent for a partial or complete autopsy after their death. Obtaining this information will improve the scientific value of the study and contribute to the safety of future research participants (12).

### Theme 6: Oversight and Accountability

The literature suggests that researchers should be especially careful when communicating with patients, physicians, other stakeholders, and the general public about regenerative medicine applications, as overly optimistic expectations might easily arise (17, 21, 22, 25, 29, 46, 52, 57, 62, 64, 67, 69, 78, 80, 81, 86, 90, 93, 94, 100) (Table 8). The ways in which research is represented in the media affects societal perspectives and frames policy debates (17, 67, 86, 100). In frontier science, of which research on bio-artificial organ transplantation is an example, researchers might wish or feel compelled to attract media attention to obtain financial support (17). However, they should refrain from inaccurate or incomplete representation of research, as this could ultimately have negative consequences for the advancement of the field and the integrity. For instance, researchers should avoid sharing findings with the press before peer review (17, 62) or could follow the ISSCR guidelines with regard to the conduct, public engagement and accountability of clinical trials (12, 16). In addition, researchers should be open to (international) collaboration between scientists, ethicists and clinicians (18, 22, 23, 25, 28, 35, 36, 38, 39, 41, 45, 50, 54, 57, 63–65, 73, 77, 81, 84–86, 89, 96, 100–102) and the conduct of interdisciplinary dialogues, involving scientists, such as engineers and biologists, but also patients, clinicians, policy makers, industry partners, ethicists, and the general public (17, 24, 29, 35, 37, 38, 46, 55, 64, 73, 80, 81, 84, 86, 90, 93) to encourage responsible innovation, and build and maintain long-term trust in research and the development of regenerative medicine applications. Adopting a similar strategy around bio-artificial organ technologies is highly desirable.

**TABLE 8:** points to consider in relation to oversight and accountability.

Oversight and accountability	Points to consider
Public awareness and patient engagement	- The information should be publicly available
	- Interdisciplinary dialogues between scientists, ethicists, patients, policy-makers, clinicians, industry partners, and the general public should be stimulated
	- Dissemination of non-peer-reviewed research results should be avoided
	- Participants should be referred to patient advocacy groups
	- Participants should have an active role in research (e.g., as active stakeholders)
Strengthening of RECs	- RECs should be expanded with experts in regenerative medicine/organ transplantation or set up advisory boards or specialized working groups to support RECs
	- Patient representatives should be invited to participate in RECs
	- Educational activities should be organized for RECs
Stimulate (data) transparency, minimize publication bias and diminish selective reporting to create long-term trust in research	- Preclinical researchers should publish negative, positive and inconclusive results
	- Researchers should pre-register clinical trials
	- Data monitoring plans should be put in place
	- Researchers, clinicians and regulators should be stimulated to collaborate
	- Guidance should be periodically revised

All research involving clinical applications of regenerative medicine must be subjected to independent RECs for approval. The main task of these oversight bodies is to ensure ethical conduct of clinical research and to protect human research participants. However, it is uncertain whether existing RECs have sufficient specific technical and clinical expertise in the fields of both organ transplantation and regenerative medicine to be able to evaluate the risks associated with bio-artificial organ transplantation trials. Multiple authors have proposed to set up specialized RECs or advisory boards with experts from various backgrounds for the evaluation of clinical trials of regenerative medicine technologies (9, 16, 19, 20, 22, 24, 28, 29, 32, 45, 46, 62–65, 67, 69, 77, 78, 80, 85, 92). These experts could assist RECs in assessing the scientific underpinnings of the clinical trial protocols and the risks of abnormal product function and proliferation (16). According to some, such specialized RECs should ideally also include lay people (21, 80). Moreover, authors recommend providing education opportunities for surgeons, researchers, nurses and ethicist in training, on the ethical aspects related to ATMPs (9, 20–22, 29, 36, 40, 45, 64, 65, 69, 70, 73, 77, 87, 92, 93).

Researchers should pre-register clinical trials and publish understandable and complete data on each step along the research pathway regardless of whether the data is positive, negative or inconclusive (12, 16, 24, 28, 29, 69, 80, 81). Being transparent about data could also inspire other researchers to go into new research directions (69).

## Discussion

In the rapidly evolving field of regenerative medicine, it is important that early-phase clinical trials are performed in a responsible and ethically acceptable way. Such trials can lead to unforeseeable serious harm for research participants, as, for instance, has occurred during early-phase clinical trials of gene therapies in the 1990s, in which research participants have died (103). Yet clinical translation of bio-artificial organ technologies has the potential to make available life-saving therapeutic products to patients suffering from end-stage organ failure and to remove the need of (life-long) immunosuppressive therapy, which has hitherto been a serious disadvantage of organ transplantation.

To our knowledge, this is the first systematic review of the literature on early-phase clinical trials in regenerative medicine, tissue engineering, cell-based therapy, bio-engineered organs, organoid transplantation, synthetic biology, and 3D bioprinting, which summarizes relevant ethical points to consider in early-phase research on transplantable bio-artificial organs. Our review reveals that a significant body of literature exists on ethical considerations around early-phase trials in the field of cell-based therapy. However, there is strikingly little literature on ethical aspects of early-phase clinical trials in the field of 3D bioprinting, and organoid transplantation. There is also little attention for ethical aspects of early-phase regenerative medicine trials in surgery; only seven papers were published in surgical journals. A further noticeable finding in this review was the paucity of empirical ethics research in the scientific fields that were included in the review: only six empirical studies were found (21, 77, 83, 93, 94, 98), three of which focussed on the perceived ethical challenges of regenerative medicine among professionals in the field (21, 77, 83), and three of which focussed on patients’ perspectives (93, 94, 98) on ethical considerations for early-phase clinical regenerative trials. Yet insight in patients’ perspectives is essential to assessing the social value of new technologies and to determining the conditions under which it should be offered to patients.

In total, six themes were identified in the literature: cell source, risk-benefit assessment, patient selection, trial design, informed consent, and oversight and accountability. We found that ethical considerations around cell sources were mentioned most often, which is consistent with an earlier review of the ethical aspects of tissue engineering by de Vries et al (38). For each of the six themes, we have distilled and discussed ethical points to consider, which can be valuable for research groups and RECs who will be setting up or evaluating early-phase clinical transplantation trials of bio-artificial organs in the future, and for health care professionals working in the field of organ transplantation with an interest in innovative technologies. Below, we would like to reflect on important points made on two themes: trial design and informed consent. These themes are underrepresented in the literature, and need specific attention before early-phase bio-artificial organ transplantation trials can be initiated, and evaluated by RECs.

First, when designing clinical trials, researchers should not focus exclusively on gathering data on clinical outcomes, but also on understanding research participants’ perspectives. Qualitative studies of patients’ perspectives can help elucidate their needs and preferences with regard to the set-up and conduct of clinical trials, the use of outcome measures, the design and performance characteristics of the product that is being developed, the type of follow-up care that will be offered, etc., so that the process of clinical development and the resulting bio-artificial organ technologies are optimally aligned with patients’ perspectives, to improve their quality of life. Also, trials should be designed such that data on long-term clinical outcomes of transplantable bioartificial organ technologies can be gathered. An exploratory survey among European tissue-engineers by Trommelmans et al. found that the majority of respondents insisted on long-term follow-up (83). Given the irreversibility of transplantation of bio-artificial organs and its potential for adverse events emerging only after a long time, long-term follow-up procedures may be essential in trials of bio-artificial organs. This requires long-term—possibly even lifelong—commitment of participants (34), and long-term trust relationships between researchers and patients. Barriers to long-term follow-up studies frequently reported include outdated contact information, lack of financial reimbursement for follow-up services, and direct and indirect costs charged to participants (104,105). Researchers in regenerative medicine could learn from prior experiences in overcoming these barriers. One such strategy is to discuss the long-term follow-up planning with participants during the informed consent procedure (106). Additional research is needed to identify barriers specific to long-term follow-up of bio-artificial organ transplantation trials, and to develop strategies for overcoming them.

Second, during the informed procedure, researchers should communicate reasonably foreseeable risks and benefits associated with participation in clinical trials. However, little guidance exists on how researchers should communicate such risk and benefits in cutting edge early-phase research (107, 108), in which there is a high degree of uncertainty surrounding these risks and benefits due to limited knowledge. There are concerns that researchers might overestimate and exaggerate the benefits in early-phase clinical trials, which is a potential source of “therapeutic misconception” (109, 110). For instance, Kimmelman et al. (110) analysed patient information and informed consent documents on risky, novel, experimental early-phase gene-transfer trials for seriously ill patients, and concluded that these were often inappropriately optimistic about the direct benefits for individual participants. The results of this study are relevant, because early-phase bio-artificial organs will also be risky and experimental. To prevent therapeutic misconception, researchers should provide realistic information to participants about the individual medical benefits and uncertainties of participation in early-phase clinical trials.

We consider it remarkable that it is often recommended, in various research fields, to use questionnaires, or extraordinarily written or oral exams, to check whether research participants have understood relevant information about clinical trial participation (16, 21, 33, 108, 110–112). It is believed that the exam approach will leave more time for the researcher, during a subsequent informed consent discussion, to focus on the aspects about which the participant’s knowledge is not yet sufficient, and tailor the process to the participant’s individual informational needs (113). However, it is unclear whether this focus on formally “testing” participants’ knowledge of (the science underlying) the trial will lead to better informed, more autonomous decisions about research participation. It may also place more responsibility or liability on research participants when—deciding about—participating in novel, possibly risky trials. Further research will be needed to understand and improve communication about risks and benefits of participation in early-phase clinical trials of bio-artificial organs.

We did not limit this review to one specific bio-artificial organ type. Instead, we developed a general list of ethical points to consider for all bio-artificial organ technologies. However, these points to consider may play out differently in specific bio-artificial organ technologies, and may vary with organ type; for instance, to a greater extent than for hearts, lungs, and livers, there are alternative (organ replacement) therapies available for pancreases or kidneys. This difference may affect risk-benefit assessment and patient selection of a clinical trial, which needs to be taken into account.

In conclusion, there is no specific ethical guidance for the safe and responsible design and conduct of early-phase clinical trials of transplantable bio-artificial organs. However, we have shown that ethical considerations from adjacent research fields may be useful for early-phase transplantable bio-artificial organs trials. In particular, the irreversibility, uncertainty of outcomes, the ethical considerations around the cell sources used to generate the product (e.g., donor cells), and the need for life-long follow-up studies makes clinical translation of bio-artificial organ technologies ethically contentious. Ethical themes that researchers and RECs should consider when designing or evaluating studies include cell source, risk-benefit assessment, patient selection, trial design, informed consent, and oversight and accountability. Patient engagement and empirical studies of patients’ perspectives on (organ-) specific bio-artificial organ technologies will be essential to realizing the social value of research and clinical translation of bio-artificial organs, and to ensuring adequate informed consent for research participation.

## VANGUARD Consortium Partners

Members of the VANGUARD consortium are as follows: Ekaterine Berishvili, Laura Mar Fonseca, Fanny Lebreton, Kevin Bellofatto, Juliette Bignard (Department of Surgery, University of Geneva, Geneva, Switzerland); Jochen Seissler, Leila Wolf-van Buerck, Mohsen Honarpisheh, Yichen Zhang, Yutian Lei, Monika Pehl (Diabetes Centre—Campus Innenstadt, Medizinische Klinik und Poliklinik IV, Klinikum der Ludwig-Maximilians-Universität München, Germany); Antonia Follenzi, Christina Olgasi, Alessia Cucci, Chiara Borsotti, Simone Assanelli (Department of Health Sciences, University of Piemonte Orientale, Novara, Italy); Lorenzo Piemonti, Antonio Citro, Silvia Pellegrini, Cataldo Pignatelli, Francesco Campo (IRCCS Ospedale San Raffaele, Diabetes Research Institute, Milano, Italy); Olivier Thaunat (Dept. Transplantation, Nephrology and Clinical Immunology, Lyon Claude Bernard University, Lyon, France); Devi Mey, Chiara Parisotto, Giovanna Rossi (European Society for Organ Transplantation, Padova, Italy); Patrick Kugelmeier, Petra Wolint, Markus Mühlemann, Karolina Pal-Kutas (Kugelmeiers AG, Erlenbach, Switzerland); Marco Cavallaro, Julia Götz, Jeanette Müller (Accelopment Switzerland Ltd.).

## Footnotes

1 VANGUARD. New generation cell therapy: bioartificial pancreas to cure type 1 diabetes. https://vanguard-project.eu/ (Accessed 1 July 2022).

## Supplementary Material

The Supplementary Materials for this article can be found online at: https://www.frontierspartnerships.org/articles/10.3389/ti.2022.10751/full#supplementary-material

Supplementary Data Sheet S1 PRISMA checklist.

Supplementary Data Sheet S2 Capsule sentence summary.

Supplementary Data Sheet S3 Search term.
