# Supplementary material for: Early-Phase Clinical Trials of Bio-Artificial Organ Technology: A Systematic Review of Ethical Issues
Source: Transpl Int. 2022 Oct 31;35:10751. doi: 10.3389/ti.2022.10751 (PMC9659568; doi:10.3389/ti.2022.10751)
Supplement: Supplementary file 1 [file DataSheet3.docx]

| **Database searched** | **via** | **Years of coverage** | **Records** | **Records after duplicates removed** |
| --- | --- | --- | --- | --- |
| Embase | Embase.com | 1971 - Present | 1549 | 1526 |
| Medline ALL | Ovid | 1946 - Present | 1024 | 267 |
| Web of Science Core Collection* | Web of Knowledge | 1975 - Present | 730 | 143 |
| Cochrane Central Register of Controlled Trials** | Wiley | 1992 - Present | 71 | 16 |
| PsycINFO | Ovid | 1806 - Present | 18 | 2 |
| Other sources: Google Scholar | | | 200 | 178 |
| **Total** | | | **3592** | **2132** |

*Science Citation Index Expanded (1975-present) ; Social Sciences Citation Index (1975-present) ; Arts & Humanities Citation Index (1975-present) ; Conference Proceedings Citation Index- Science (1990-present) ; Conference Proceedings Citation Index- Social Science & Humanities (1990-present) ; Emerging Sources Citation Index (2015-present)
** Manually deleted abstracts from trial registries

**Embase.com**

('ethics'/exp OR 'bioethics and professional ethics'/de OR 'moral status'/de OR 'patient right'/exp OR 'morality'/de OR 'social aspect'/de OR 'ethicist'/de OR 'patient selection'/de OR (ethic* OR unethic* OR bioethic* OR bio$ethic* OR ((moral OR morale OR morals OR morales) NEAR/3 (status* OR stand* OR polic* OR medic* OR concern* OR obligation* OR duty OR duties)) OR ((patient OR patients) NEXT/1 (right OR rights OR autonom*)) OR confidentialit* OR informed-consent* OR misconception* OR morality OR immoral* OR equipois* OR ((social) NEAR/3 (aspect* OR factor*)) OR (responsible* NEAR/3 condition*) OR (risk* NEAR/3 assess*)):ab,ti,kw) **AND** ('bioengineering'/de OR 'cell engineering'/de OR 'biomedical engineering'/de OR 'bioartificial organ'/exp OR 'engineered tissue'/exp OR 'bioprinting'/exp OR 'tissue engineering'/de OR 'regenerative medicine'/exp OR 'organoid'/exp OR 'organoid culture'/de OR (bioengineer* OR bio*-engineer* OR tissue-engineer* OR human-engineer* OR emerg*-technolog* OR ((bioartificial* OR bio*-artificial* OR engineer* OR regenerat* OR 3D OR 3-D OR threedimension* OR three-dimension* OR print* OR bioprint*) NEAR/3 (organ* OR tissue* OR liver* OR kidney* OR pancreas* OR heart* OR cardia* OR bladder* OR hepat* OR renal* OR lung* OR pulmonar*)) OR regenerat*-medicine* OR organoid* OR ((mini) NEAR/3 (organ OR organs OR brain*)) OR colonoid* OR enteroid* OR (organ* NEAR/1 dish*)):ab,ti,kw) **AND** ('transplantation'/exp OR 'clinical trial'/exp OR 'translational research'/de OR (transplant* OR bench-to-bedside* OR first-in-human* OR ((clinical*) NEAR/3 (translat* OR research* OR trial*)) OR ((translational*) NEAR/3 (medicin* OR research*))):ab,ti,kw) *NOT ((animal/exp OR animal*:de OR nonhuman/de) NOT ('human'/exp))* *NOT [conference abstract]/lim*

**Medline (Ovid)**

(exp Ethics/ OR exp Patient Rights/ OR Morals/ OR ethics.fs. OR Patient Selection/ OR (ethic* OR unethic* OR bioethic* OR bio$ethic* OR ((moral OR morale OR morals OR morales) ADJ3 (status* OR stand* OR polic* OR medic* OR concern* OR obligation* OR duty OR duties)) OR ((patient OR patients) ADJ (right OR rights OR autonom*)) OR confidentialit* OR informed-consent* OR misconception* OR morality OR immoral* OR equipois* OR ((social) ADJ3 (aspect* OR factor*)) OR (responsible* ADJ3 condition*) OR (risk* ADJ3 assess*)).ab,ti,kf.) **AND** (Bioengineering/ OR Cell Engineering/ OR Biomedical Engineering/ OR Bioartificial Organs/ OR Bioprinting/ OR Tissue Engineering/ OR Regenerative Medicine/ OR Organoids/ OR (bioengineer* OR bio*-engineer* OR tissue-engineer* OR human-engineer* OR emerg*-technolog* OR ((bioartificial* OR bio*-artificial* OR engineer* OR regenerat* OR 3D OR 3-D OR threedimension* OR three-dimension* OR print* OR bioprint*) ADJ3 (organ* OR tissue* OR liver* OR kidney* OR pancreas* OR heart* OR cardia* OR bladder* OR hepat* OR renal* OR lung* OR pulmonar*)) OR regenerat*-medicine* OR organoid* OR ((mini) ADJ3 (organ OR organs OR brain*)) OR colonoid* OR enteroid* OR (organ* ADJ1 dish*)).ab,ti,kf.) **AND** (exp Transplantation/ OR transplantation.fx. OR exp Clinical Trial/ OR Translational Medical Research/ OR (transplant* OR bench-to-bedside* OR first-in-human* OR ((clinical*) ADJ3 (translat* OR research* OR trial*)) OR ((translational*) ADJ3 (medicin* OR research*))).ab,ti,kf.) *NOT (exp animals/ NOT humans/)* *NOT (letter* OR news OR comment* OR editorial* OR congres* OR abstract* OR book* OR chapter* OR dissertation abstract*).pt.*

**Web of Science**

TS=(((ethic* OR unethic* OR bioethic* OR bio$ethic* OR ((moral OR morale OR morals OR morales) NEAR/2 (status* OR stand* OR polic* OR medic* OR concern* OR obligation* OR duty OR duties)) OR ((patient OR patients) NEAR/1 (right OR rights OR autonom*)) OR confidentialit* OR informed-consent* OR misconception* OR morality OR immoral* OR equipois* OR ((social) NEAR/2 (aspect* OR factor*)) OR (responsible* NEAR/2 condition*) OR (risk* NEAR/2 assess*))) **AND** ((bioengineer* OR bio*-engineer* OR tissue-engineer* OR human-engineer* OR emerg*-technolog* OR ((bioartificial* OR bio*-artificial* OR engineer* OR regenerat* OR 3D OR 3-D OR threedimension* OR three-dimension* OR print* OR bioprint*) NEAR/2 (organ* OR tissue* OR liver* OR kidney* OR pancreas* OR heart* OR cardia* OR bladder* OR hepat* OR renal* OR lung* OR pulmonar*)) OR regenerat*-medicine* OR organoid* OR ((mini) NEAR/2 (organ OR organs OR brain*)) OR colonoid* OR enteroid* OR (organ* NEAR/1 dish*))) **AND** ((transplant* OR bench-to-bedside* OR first-in-human* OR ((clinical*) NEAR/2 (translat* OR research* OR trial*)) OR ((translational*) NEAR/3 (medicin* OR research*)))) NOT ((animal* OR rat OR rats OR mouse OR mice OR murine OR dog OR dogs OR canine OR cat OR cats OR feline OR rabbit OR cow OR cows OR bovine OR rodent* OR sheep OR ovine OR pig OR swine OR porcine OR veterinar* OR chick* OR zebrafish* OR baboon* OR nonhuman* OR primate* OR cattle* OR goose OR geese OR duck OR macaque* OR avian* OR bird* OR fish*) NOT (human* OR patient* OR women OR woman OR men OR man))) *AND DT=(Article OR Review OR Early Access)*

**Cochrane Central**

((ethic* OR unethic* OR bioethic* OR bio$ethic* OR ((moral OR morale OR morals OR morales) NEAR/3 (status* OR stand* OR polic* OR medic* OR concern* OR obligation* OR duty OR duties)) OR ((patient OR patients) NEXT/1 (right OR rights OR autonom*)) OR confidentialit* OR informed NEXT consent* OR misconception* OR morality OR immoral* OR equipois* OR ((social) NEAR/3 (aspect* OR factor*)) OR (responsible* NEAR/3 condition*) OR (risk* NEAR/3 assess*)):ab,ti,kw) **AND** ((bioengineer* OR bio* NEXT engineer* OR tissue NEXT engineer* OR human NEXT engineer* OR emerg* NEXT technolog* OR ((bioartificial* OR bio* NEXT artificial* OR engineer* OR regenerat* OR 3D OR 3 NEXT D OR threedimension* OR three NEXT dimension* OR print* OR bioprint*) NEAR/3 (organ* OR tissue* OR liver* OR kidney* OR pancreas* OR heart* OR cardia* OR bladder* OR hepat* OR renal* OR lung* OR pulmonar*)) OR regenerat* NEXT medicine* OR organoid* OR ((mini) NEAR/3 (organ OR organs OR brain*)) OR colonoid* OR enteroid* OR (organ* NEAR/1 dish*)):ab,ti,kw) *NOT “conference abstract”:pt*

**PsycINFO**

(exp Ethics/ OR exp Client Rights/ OR exp Morals/ OR Patient Selection/ OR (ethic* OR unethic* OR bioethic* OR bio$ethic* OR ((moral OR morale OR morals OR morales) ADJ3 (status* OR stand* OR polic* OR medic* OR concern* OR obligation* OR duty OR duties)) OR ((patient OR patients) ADJ (right OR rights OR autonom*)) OR confidentialit* OR informed-consent* OR misconception* OR morality OR immoral* OR equipois* OR ((social) ADJ3 (aspect* OR factor*)) OR (responsible* ADJ3 condition*) OR (risk* ADJ3 assess*)).ab,ti.) **AND** ((bioengineer* OR bio*-engineer* OR tissue-engineer* OR human-engineer* OR emerg*-technolog* OR ((bioartificial* OR bio*-artificial* OR engineer* OR regenerat* OR 3D OR 3-D OR threedimension* OR three-dimension* OR print* OR bioprint*) ADJ3 (organ* OR tissue* OR liver* OR kidney* OR pancreas* OR heart* OR cardia* OR bladder* OR hepat* OR renal* OR lung* OR pulmonar*)) OR regenerat*-medicine* OR organoid* OR ((mini) ADJ3 (organ OR organs OR brain*)) OR colonoid* OR enteroid* OR (organ* ADJ1 dish*)).ab,ti.) **AND** (Organ Transplantation/ OR exp Clinical Trials/ OR (transplant* OR bench-to-bedside* OR first-in-human* OR ((clinical*) ADJ3 (translat* OR research* OR trial*)) OR ((translational*) ADJ3 (medicin* OR research*))).ab,ti.) *NOT (letter* OR news OR comment* OR editorial* OR congres* OR abstract* OR book* OR chapter* OR dissertation abstract*).pt.*

**Google Scholar**

Ethics|ethical|unethical|bioethics|moral|morality|immoral bioengineering|bio-engineering|“bioartificial|bio-artificial|3D|3-D|threedimensional|print|bioprint organ|tissue” transplantation|“clinical translation|research|trial”
