# Supplementary material for: Early-Phase Clinical Trials of Bio-Artificial Organ Technology: A Systematic Review of Ethical Issues
Source: Transpl Int. 2022 Oct 31;35:10751. doi: 10.3389/ti.2022.10751 (PMC9659568; doi:10.3389/ti.2022.10751)
Supplement: Supplementary file 3 [file DataSheet2.docx]

**Capsule Sentence Summary**

It is anticipated that first-in-human clinical trials will be conducted to test the safety and efficacy of bio-artificial transplantable organs in human recipients. This systematic review presents relevant ethical points to consider.
